# The Physiological Basis of High-Frequency Oscillatory Ventilation and Current Evidence in Adults and Children: A Narrative Review

**DOI:** 10.3389/fphys.2022.813478

**Published:** 2022-04-26

**Authors:** Andrew G. Miller, Herng Lee Tan, Brian J. Smith, Alexandre T. Rotta, Jan Hau Lee

**Affiliations:** ^1^ Duke University Medical Center, Respiratory Care Services, Durham, NC, United States; ^2^ KK Women’s and Children’s Hospital, Children’s Intensive Care Unit, Singapore, Singapore; ^3^ University of California, Davis, Respiratory Care Services, Sacramento, CA, United States; ^4^ Duke University Medical Center, Division of Pediatric Critical Care Medicine, Durham, NC, United States; ^5^ Duke-NUS Medical School, Singapore, Singapore

**Keywords:** mechanical ventilation (lung protection) strategy, high-frequency ventilation with oscillations, high-frequency ventilation, children, ARDS, review (article), lung injury

## Abstract

High-frequency oscillatory ventilation (HFOV) is a type of invasive mechanical ventilation that employs supra-physiologic respiratory rates and low tidal volumes (V_T_) that approximate the anatomic deadspace. During HFOV, mean airway pressure is set and gas is then displaced towards and away from the patient through a piston. Carbon dioxide (CO_2_) is cleared based on the power (amplitude) setting and frequency, with lower frequencies resulting in higher V_T_ and CO_2_ clearance. Airway pressure amplitude is significantly attenuated throughout the respiratory system and mechanical strain and stress on the alveoli are theoretically minimized. HFOV has been purported as a form of lung protective ventilation that minimizes volutrauma, atelectrauma, and biotrauma. Following two large randomized controlled trials showing no benefit and harm, respectively, HFOV has largely been abandoned in adults with ARDS. A multi-center clinical trial in children is ongoing. This article aims to review the physiologic rationale for the use of HFOV in patients with acute respiratory failure, summarize relevant bench and animal models, and discuss the potential use of HFOV as a primary and rescue mode in adults and children with severe respiratory failure.

## Introduction

Acute respiratory distress syndrome (ARDS) is a disease of acute onset characterized by significant hypoxemia and typical radiographic findings that affects both children and adults, and is an important cause of morbidity and mortality worldwide (Force et al., 2012; Pediatric Acute Lung Injury Consensus Conference, 2015; Thompson et al., 2017; Khemani et al., 2019). Whether pulmonary injury is the result of a direct (e.g., pneumonia, smoke inhalation, lung contusion) or indirect (e.g., sepsis, blood transfusion) insult, disease distribution in ARDS is heterogeneous, with more severe involvement of the dependent and relative sparing of the non-dependent lung regions (Ware and Matthay, 2000; Gattinoni et al., 2017b). This heterogeneous distribution of lung disease poses a challenge to the clinician instituting positive pressure ventilation, as different areas of the lung will have vastly different compliance and resistance. Non-dependent or uninjured alveoli (with better compliance) are at risk of overdistension, while dependent or injured alveoli (with worse compliance) are at risk of de-recruitment and repeated opening and closing with each respiratory cycle (Ware and Matthay, 2000; Gattinoni et al., 2017b).

**FIGURE 1:**
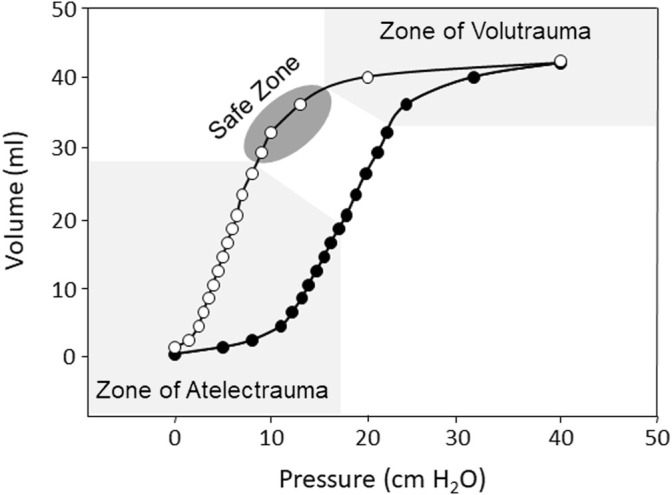
Representation of the inspiratory (black circles) and expiratory (white circles) static pressure-volume curves from a rabbit saline lavage model of ARDS showing hysteresis between the inspiratory and expiratory curves, the zone of volutrauma and atelectrauma (light gray), and the theoretical safe zone of ventilation (dark gray).

When precisely employed, mechanical ventilation (MV) is a life-saving intervention, yet care must be taken to avoid ventilator-induced lung injury (VILI) (Tremblay and Slutsky, 2006; Beitler et al., 2016). Several factors have been identified as contributors to VILI. These include injury from the delivery of excessive pressure (barotrauma or stress) or tidal volume (V_T_) (volutrauma or strain), injury from the cyclic opening and closing of alveoli (atelectrauma), toxicity caused by high inspired fraction of inspired oxygen (FiO_2_), and injury resulting from cytokine release that can affect end-organ function (biotrauma) (Tremblay and Slutsky, 2006; Beitler et al., 2016). The landmark ARMA trial comparing mechanical ventilation with a tidal volume of 12 ml/kg to 6 ml/kg (both calculated using predicted body weight) found a significant mortality benefit with the application of a lower V_T_ strategy and confirmed the role of high V_T_ in VILI (Acute Respiratory Distress Syndrome et al., 2000). This study renewed interest in high-frequency oscillatory ventilation (HFOV) as an ultra-protective lung protective strategy capable of delivering very low V_T_. MV strategies aimed at avoiding VILI are termed “lung protective,” and generally operate in a theoretical “safe zone” on the deflating limb of the static pressure/volume curve (Figure 1).

A study by Amato et al., published in 2015, elegantly illustrated the direct association between driving pressure (plateau pressure minus measured PEEP) and mortality in ARDS (Amato et al., 2015). Subsequent studies have also implicated driving pressure as a key variable that is associated with mortality in ARDS (Guerin et al., 2016; Goligher et al., 2021). Recent studies have highlighted the role of total energy delivered during each tidal breath to be an important factor in VILI (Gattinoni et al., 2017a). This concept, referred to as mechanical power, incorporates all mechanical ventilator settings, including respiratory rate, driving pressure, PEEP, and inspiratory flow (Gattinoni et al., 2017a). Mechanical power is an appealing concept because it accounts for the energy required to distend the lung, move gas, and maintain lung volume (Gattinoni et al., 2017a). The lung injury resultant from energy transmission from the various elements that determine mechanical power is termed ergotrauma. Indeed, mechanical power and the resultant ergotrauma have been directly associated with unfavorable outcomes in both adults and children with ARDS (Costa et al., 2021; Bhalla et al., 2022). A recent study found only respiratory rate and driving pressure to be independent predictors of mortality among variables included in the calculation for mechanical power (Costa et al., 2021). The simplified equation using these variables for estimation of mechanical power [(4 × ΔP) + RR] had a similar predictive value for mortality as mechanical power calculated using the more complex original method (Costa et al., 2021). Thus, mechanical power is an intriguing concept, but its utility as a modifiable parameter needs confirmation in a clinical trial.

**FIGURE 2:**
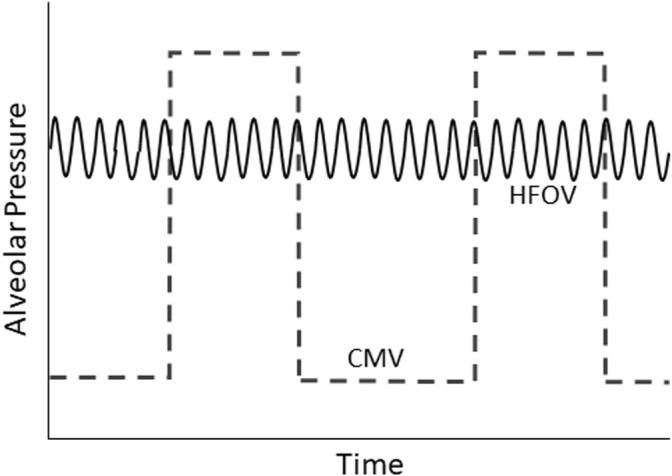
Schematic representation of alveolar pressure over time during conventional mechanical ventilation (CMV) and high-frequency oscillatory ventilation (HFOV).

Although lung protective ventilation can certainly be achieved through carefully conducted conventional mechanical ventilation (CMV), the lower V_T_, lower alveolar pressure swings, and higher mean airway pressure (mPaw) generally employed during various forms of high frequency ventilation make these modalities theoretically well suited for lung protection (Figure 2). There are four types of high frequency ventilation in clinical use: HFOV, high frequency jet ventilation, high-frequency percussive ventilation, and high frequency flow interruption (Keszler et al., 2015; Miller A. G. et al., 2021). This review will focus on the physiologic rationale for the use of HFOV in patients with acute respiratory failure, summarize data from relevant bench and animal models, and discuss the potential use of HFOV as a primary and rescue mode in adults and children with severe respiratory failure.

### Theory of HFOV Operation

HFOV is a form of MV that uses a constant distending pressure, usually reported as the mPaw, coupled with sinusoidal or square flow oscillations at supra-physiologic respiratory frequencies (Rettig et al., 2015; Miller A. G. et al., 2021). Respiratory frequencies used in clinical practice range from 5 to 15 Hz (i.e., 300 to 900 breaths per minute) with a small delivered V_T_, generally around 1–3 ml/kg, or lower than the anatomic dead space (Rettig et al., 2015; Miller A. G. et al., 2021). The constant distending pressure allows for alveolar recruitment while avoiding repetitive opening and closing of alveoli (atelectrauma), and has been shown to improve oxygenation (Rettig et al., 2015; Meyers et al., 2019). HFOV may also decrease the occurrence of volutrauma and barotrauma (Rettig et al., 2015).

**FIGURE 3:**
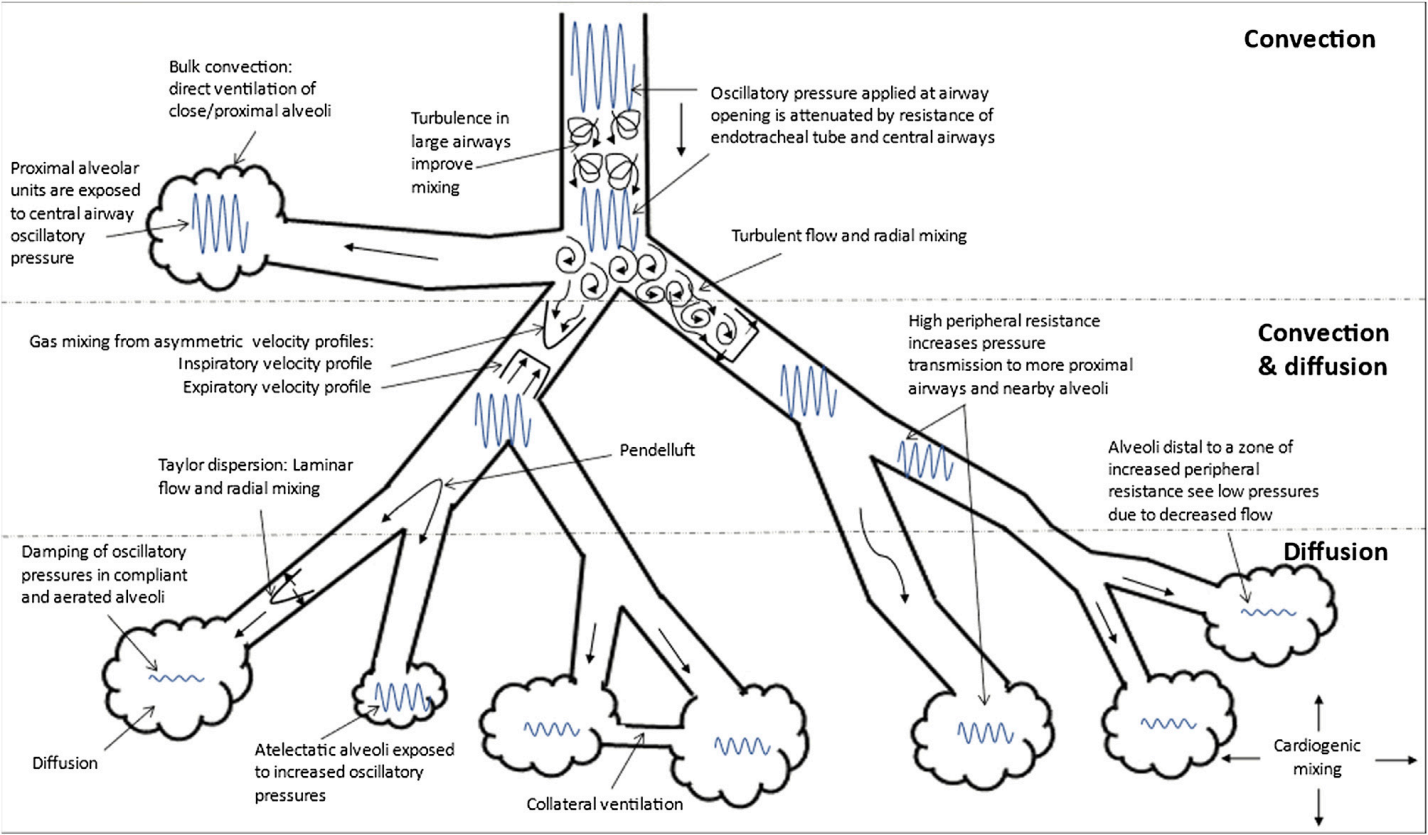
Gas Transport Mechanisms During High Frequency Oscillatory Ventilation (HFOV). Adapted from references: (Slutsky and Drazen, 2002; Pillow, 2005). The gas exchange mechanisms that function in each region (convection, convection and diffusion and diffusion alone) are shown. The various mechanisms that contribute to gas transport during HFOV are: 1) turbulence in large airways producing improved mixing; 2) bulk convection (direct ventilation of close alveoli); 3) turbulent flow with lateral convective mixing; 4) pendelluft (asynchronous flow among alveoli due to asymmetries in airflow impedance); 5) asymmetric inspiratory and expiratory velocity profiles (gas mixing due to velocity profiles that are axially asymmetric resulting in streaming of fresh gas toward alveoli along the inner wall of the airway and the streaming of alveolar gas away from the alveoli along the outer wall); 6) Taylor dispersion (laminar flow with lateral transport by diffusion); 7) collateral ventilation through non-airway connections between neighboring alveoli; and 8) cardiogenic mixing (rhythmic, pulsatile nature of the heart conferring a mixing of gases). The extent to which the oscillatory waveform is attenuated is also shown in this figure. Atelectatic alveoli will experience higher oscillatory pressure and lesser damping compared to normally aerated alveoli. Increase in peripheral resistance, other the other hand increase pressure transmission to more proximal airways and nearby alveoli such that alveoli distal to this zone of increased peripheral resistance experience lower pressures due to decreased flow.

HFOV differs from CMV and high frequency jet ventilation in that both inspiration and expiration are active (Miller A. G. et al., 2021; Miller A. G. et al., 2021). Oxygenation and ventilation are fairly independent during HFOV with oxygenation being controlled by FiO_2_ and mPaw while ventilation is controlled by V_T_ (amplitude) and frequency (f) (Kneyber et al., 2012; Miller A. G. et al., 2021). Various mechanisms contribute to gas exchange during HFOV; these include gas flow turbulence in large airways, bulk convection, turbulent flow with radial mixing, pendelluft, asymmetric inspiratory and expiratory velocity profiles, Taylor dispersion, collateral ventilation, and cardiogenic mixing (Slutsky and Drazen, 2002; Pillow, 2005) (Figure 3).

### HFOV Mechanics

The mechanism of gas exchange during HFOV varies according to the method of oscillation generation, attenuation of the pressure waveform, and efficiency of volume delivery (Pillow et al., 2001; John et al., 2014). HFOV produces biphasic pressure waveforms and diverts fresh bias flow to the patient at frequencies greater than 3 Hz (Keszler et al., 2015). The matching of positive and negative pressure deflections results in both inspiratory and expiration phases being active and can be achieved by a linear motor piston pump, an electromagnetically-driven vibrating diaphragm device, or an expiratory venturi jet (Keszler et al., 2015).

HFOV can be delivered via dedicated HFOV ventilator [e.g., Sensormedics 3100A and 3100B (Carefusion, Yorba Linda, California, United States)], or hybrid ventilators. Available HFOV ventilators and their mechanism of action are summarized in Table 1. Dedicated HFOV and hybrid ventilators differ in how they generate oscillations, ability to measure V_T_, availability of volume-targeted mode, range of settings for flow, pressure amplitude, frequency, and I:E ratio (Table 1) (Grazioli et al., 2015; Keszler et al., 2015; Tingay et al., 2015).

**TABLE 1:** Characteristics of various high frequency oscillatory ventilators.

	Sensormedics 3100A	Sensormedics 3100B	Metran R100	Fabian HFO	Leoni plus	Stephan sophie	Sle 5000	Sle 6000	Drager babylog VN500
Principle of operation	Piston HFOV						Non-piston HFOV		
	Oscillations generated by electro-magnetic diaphragm moving back and forth, similar to a permanent magnet loudspeaker		Oscillations provided from a diaphragm on the anterior side of the ventilator which is driven by a rotary valve mechanism	Oscillations generated by piston pushing a diaphragm back and forth (diaphragm principle)	Oscillations generated by piston pushing a diaphragm back and forth (diaphragm principle)	Valve oscillator with active exhalation	Oscillations achieved by active exhalation, and rapid cycling of the forward and reverse jets		Oscillations generated by intermittent negative pressure generated by a venturi effect of the high-flow jet injector at the expiratory valve causing exhalation
Mode	HFOV only		CMV and HFOV	CMV and HFOV	CMV and HFOV	CMV and HFOV	CMV and HFOV		CMV and HFOV
Patient population	All, 3100A for patients <35 kg		CMV: VCV for neonates >8 kg HFOV: For infants, pediatrics and adults (upper limit of body weight not specified)	Up to 30 kg	Up to 30 kg	Up to 25 kg	SLE 5000: Up to 20 kg SLE 6000: Up to 30 kg		CMV: neonates, pediatrics and adults HFOV: up to 10 kg
Volume-targeted mode	No		No	Yes	Yes	Yes	Yes		Yes
V_T_ monitoring	No		V_T_ monitoring during CMV only	Hot-wire anemometer	Hot-wire anemometer	Hot-wire anemometer	Hot-wire anemometer		Hot-wire anemometer
Flow	0–40 L/min	0–60 L/min	10–40 L/min	5–20 L/min (neonatal, ≤10 kg)	7 L/min	0.2–10 L/min	8 L/min		2–30 L/min
				5–30 L/min (pediatric, 10–30 kg)					
Pressure amplitude setting range	1–90 cm H_2_O		Pressure amplitude is a measured value. Stroke volume is set instead Stroke volume of 2–350 ml. (5 Hz: 14–350 ml. 10 Hz: 6–160 ml. 15 Hz: 2–100 ml)	4–80 cm H_2_O	5–100 cm H_2_O	5–100% (depth of oscillations expressed as percentage swing—peak to trough—around MAP)	4–160 cm H_2_O	4–180 cm H_2_O	5–90 cm H_2_O
			5–100% (depth of oscillations expressed as percentage swing—peak to trough—around MAP)						
Mean airway pressure setting range	3–45 cm H_2_O	3–55 cm H_2_O	5–60 cm H_2_O	5–50 cm H_2_O	0–40 cm H_2_O	0–30 cm H_2_O	0–45 cm H_2_O		5–50 cm H_2_O
Frequency setting range	3–15 Hz		5–15 Hz	5–20 Hz	5–20 Hz	5–15 Hz	3–20 Hz		5–20 Hz
Inspiratory: Expiratory ratio	1:1 and 1:2		1:1	1:1 to 1:3	1:1 to 1:3	1:1 to 1:2	1:1 to 1:3		1:1 to 1:3

Oxygenation during HFOV is directly correlated with alveolar recruitment (i.e. alveolar surface area available for gas exchange), which is controlled largely by mPaw (Meyers et al., 2019). Optimizing mPaw strikes a balance between avoidance of de-recruitment and overdistension (Meyers et al., 2019).

Ventilation efficiency during HFOV (Q) can be expressed as (Slutsky et al., 1980; Boynton et al., 1989; Pillow, 2005):

$${\text{Q }=\text{ f x V}}_{T}^{2}$$

V_T_ is inversely proportional to frequency and directly proportional to amplitude (Sedeek et al., 2003; Miller A. G. et al., 2021). Therefore, the higher the frequency, the lower the V_T_; the higher the amplitude, the higher the V_T_ (Sedeek et al., 2003; Miller A. G. et al., 2021). Similarly, a higher inspiratory time percentage results in increased ventilation due to higher V_T_ (Miller A. G. et al., 2021). However, with the commonly used inspiratory time percentage of 33% [inspiratory (I) to expiratory (E) ratio of 1:2], the effect of decreasing amplitude has a greater impact on decreasing V_T_ compared to increasing frequency (Sedeek et al., 2003). In contrast, with the inspiratory percentage set at 50% (I:E of 1:1), changes in frequency have a more pronounced effect on delivered V_T_ compared to changes in amplitude (Sedeek et al., 2003). Ventilation is also affected by endotracheal tube (ETT) length and diameter, presence of a leak around the ETT, airway resistance, and respiratory system compliance (Van de Kieft et al., 2005). V_T_ has been demonstrated in theoretical models, animals, and humans to have a greater effect on gas exchange than frequency during HFOV (Boynton et al., 1989; Pillow, 2005).

Diameter and length of the ETT affect V_T_ delivery during HFOV. An increase in resistance is observed as ETT diameter deceases and as the length of the ETT increases, resulting in smaller delivered V_T_ (Pillow et al., 2002; Van de Kieft et al., 2005; Custer et al., 2011). Creation of a cuff leak increases inhaled V_T_ but reduces exhaled V_T_, and moves the source of fresh gas more distally towards the tip of the ETT (Van de Kieft et al., 2005; Van de Kieft et al., 2005; Bostick et al., 2012a). The Sensormedics 3100B has been shown to generate negative pressure during the exhalation and entrain CO_2_ into the inspiratory limb of the circuit, which can be reduced with the creation of cuff leak (Bostick et al., 2012a). Lastly, in a bench and clinical study, V_T_ was found to be higher and CO_2_ elimination greater when the piston position for the Sensormedics 3100A was displaced towards the left compared to when the piston was in the center or displaced to the right for any given amplitude, frequency and inspiratory time (Hamel et al., 2005).

Airway resistance and compliance affect CO_2_ clearance during HFOV (Kneyber et al., 2012). The amplitude of the tracheal oscillatory pressure waveform decreases with increasing peripheral resistance, resulting in reduction of transmission of pressure over the airways to the alveoli (van Genderingen et al., 2001; Pillow, 2005; Kneyber et al., 2012). The opposite happens with reduced compliance, in which there is increase in pressure transmission to the alveoli and bronchi (van Genderingen et al., 2001; Pillow, 2005; Kneyber et al., 2012). Hence pressure transmission to the alveoli is the highest in patients with low compliance and low resistance.

Spontaneous breathing during HFOV may improve oxygenation and ventilation (van Heerde et al., 2009; van Heerde et al., 2010; Kneyber et al., 2012). However, due to limitations on maximal bias flow delivered during HFOV, spontaneous breathing may be challenging for older children who have higher inspiratory flow demands than the bias flow being delivered by the ventilator, resulting in an imposed increased work of breathing (van Heerde et al., 2006; Kneyber et al., 2012; Bordessoule et al., 2018). Hence, while neonates often tolerate spontaneous breathing during HFOV without the need for deep sedation or neuromuscular blockade, that is generally not the case for children and adults (Kneyber et al., 2012; Bordessoule et al., 2018).

While the physiology of gas exchange is similar between adults and children, some important differences exist between the two. Infants and children have shorter time constants, with resulting differing HFOV setting requirements. In general, children are managed with higher frequencies compared to adults, although significant variation in management exists (Arnold et al., 2000; de Jager et al., 2019). Thus, an infant on HFOV may be managed on a frequency of 10–12 Hz while an adult or larger child may require a frequency of 5–8 Hz. However, this will vary depending on the HFOV strategy used, severity of lung injury, and lung mechanics.

In clinical practice, oxygenation is managed by adjusting the mPaw or the fraction of inspired oxygen (FiO_2_). If oxygenation is below goal, the mPaw generally is increased in 1–2 cmH_2_O until oxygenation improves. Some centers may also employ a recruitment maneuver (e.g., rapid increase in mPaw by 10–15 cmH_2_O for 30–60 s) to improve oxygenation; although this strategy can result in hemodynamic compromise. Other strategies include incremental recruitment/decruitment maneuvers to find the optimal mPaw (de Jager et al., 2019). Paradoxically, excessive mPaw may result in worsening of oxygenation due to overdistension and in some cases a trial of decreased mPaw may be warranted. If oxygenation is above goal and the FiO_2_ is already within a non-toxic range (i.e., ≤0.50) mPaw generally is decreased in steps of 1–2 cmH_2_O as part of a weaning strategy. The frequency of adjustments is dependent upon patient characteristics and local practice.

Ventilation is controlled by the pressure amplitude and frequency (respiratory rate). To increase ventilation and decrease PaCO_2_, the amplitude can be increased or the frequency decreased. Different strategies are used depending on local practice, with some centers using a fixed frequency (higher frequencies being most protective) while adjusting amplitude to affect PaCO_2_, some maximize amplitude and use frequency as the main variable affecting ventilation, and others use a combination of the two strategies. The ideal method is unknown as direct comparisons have not been performed. Similar to oxygenation, overdistension may impair ventilation while lung recruitment can result in increased CO_2_ clearance without changes to frequency or amplitude.

### Evidence for HFOV

#### Bench Models

Bench models of HFOV have demonstrated that adjustments to frequency have a larger effect on delivered V_T_ than changes to amplitude (Van de Kieft et al., 2005; Wong et al., 2017). For instance, a 2 Hz increase in frequency results in a 21% decrease in V_T_, while a 10 cm H_2_O increase in pressure amplitude is necessary for an equivalent decrease in V_T_ (Hager et al., 2007). When studied in patients, increasing frequency by 2 Hz decreased V_T_ by 23%, while increasing amplitude by 10 cmH_2_O resulted in a 5.6% increase in V_T_ (Hager et al., 2007). V_T_ delivery also decreases as ETT size is reduced, due to the higher resistance across the smaller tube. Increasing bias flow from 20 to 30 L/min increases V_T_ by 11% but this relationship is not linear; further increasing the bias flow from 30 to 40 L/min only results in a 3% increase in V_T_ (Hager et al., 2007). Increasing bias flow has been shown to improve CO_2_ clearance up to 30 L/min (Nagano et al., 2018). CO_2_ clearance is most efficient with the R100 at 50% inspiratory time and least efficient with the 3100B at an inspiratory time 33% (Yumoto et al., 2019).

In the R100 ventilator with a 50% inspiratory time, V_T_ was lower with smaller ETTs and higher frequencies (Hirao et al., 2009). When comparing V_T_ delivery between the R100 and 3100B, V_T_ was higher at similar settings with the R100 but comparable at 9 Hz and when inspiratory time was set at 50% on the 3100B (Iguchi et al., 2010). Other studies have found similar differences in V_T_ delivery. s(Custer et al., 2011). Pressure delivery is attenuated when I:E is set at 1:2 using the 3100B (Hirayama et al., 2014).

Pendelluft has been observed between lung units, largely occurring during expiration (Lee et al., 2006). An adult bench model found negative pressure within the inspiratory limb of the circuit and CO_2_ rebreathing that became detectable when amplitude was >70 cmH_2_O and continued to increase as amplitude increased. CO_2_ rebreathing was eliminated by instituting an ETT cuff leak and increasing bias flow (Bostick et al., 2012b). Pressure amplitude significantly decreases throughout the respiratory system and this pressure attenuation is directly proportional to resistance increase and inversely proportional to compliance (Rozanek et al., 2012).

A computational model found that the resonant frequency of the non-injured lung is 8 Hz while the injured lung has a resonant frequency of 17 Hz (Herrmann et al., 2016). Due to the heterogeneous nature of disease distribution in most patients, individual lung sections may have different optimal frequencies, making the frequency section challenging in clinical practice (Herrmann et al., 2016).

### Animal Studies

Early animal models provided significant insights into the mechanisms of HFOV and were well summarized in a prior review (Kacmarek and Malhotra, 2005). These early models indicated that HFOV settings required to provide normocapnia were determined by (V_T_*f) n = 0.73*W*(V_T_/V_L_)^−1.1^ and that the f* V_T_ during HFOV was higher than during CMV (Kacmarek and Malhotra, 2005). Early experiments also found that partial pressure of arterial CO_2_ (PaCO_2_) was held constant at different I:E ratio if mean lung volume and V_T_ were held constant up to a frequency of 9 Hz. This is due to gas velocity profiles being unaffected by bulk flow rate. Additional models found that as V_T_ increases, gas transport changes from dispersion (less efficient) to bulk gas flow (convection). Another study found that V_T_ *f remained constant with frequencies of 3, 6, and 9 Hz. Regional gas distribution was most homogenous at 9 Hz compared to CMV and to lower frequencies. The ability to adequately exchange gas with HFOV was demonstrated by Bohn et al., in 1980 (Bohn et al., 1980). Another seminal study in HFOV found that V_T_ *f was not the only determinant of CO_2_ clearance and that Taylor laminar and turbulent dispersion, pendelluft and asymmetrical velocity profiles were also factors. Early animal studies also showed that V_T_ was directly related to amplitude and inversely proportional to frequency (Hz) (Kacmarek and Malhotra, 2005).

Studies using animal models have consistently found improved oxygenation with HFOV (Meyer et al., 2006; Ronchi et al., 2011; Li et al., 2015; Fioretto et al., 2019). However, HFOV did not offer an advantage over protective CMV for various markers of lung injury, both in a rabbit model of lung lavage (Rotta et al., 2001) or acid aspiration (Allardet-Servent et al., 2008). In an *ex-vivo* rabbit model of air leak, both stroke volume and mPaw influenced air leak flow, but mPaw appeared to be the main independent driver of air leak (Liu et al., 2007). Circuit disconnection was evaluated in a tween pig model of lung injury showing that disconnections resulted in worsening compliance and increase in FiO_2_ requirement, with these effects persisting over time (Kubiak et al., 2010). A study evaluating stepwise decreases in mPaw in a tween pig model of lung injury found that the titrated HFOV group had more atelectasis, fibrin, congestion, PMN invasion, and regional overdistension (Maggio et al., 2010).

In a rat model of saline lavage or lung injury from lipopolysaccharide administration, HFOV use resulted in decreased lung inflammation compared to CMV with low PEEP, but was similar to the decrease in inflammation observed in CMV with optimized PEEP (Krebs et al., 2010). A saline lavage model of sheep found that transpulmonary pressure was lowest at 9 Hz, which coincided with the lowest degree of lung inflammation (Liu et al., 2013). A porcine model of oleic acid lung injury found greater lung strain at lower frequency, with the lowest strain noted at a frequency of 20 Hz (Herrmann et al., 2020).

HFOV and protective MV have similar hemodynamic effects (Roosens et al., 2006). HFOV improves oxygenation without significant depression of cardiac function (Nakagawa et al., 2007). It also did not have deleterious effects on cerebral and systemic hemodynamics in a porcine model when mPaw was set 5 cmH_2_O above the CMV mPaw (Heuer et al., 2012). A porcine model of saline lavage found that mean arterial pressure and cardiac output increased during a decremental mPaw maneuver while central venous and wedge pressure decreased (Liu et al., 2020).

A study in pigs using saline lavage to cause severe lung injury found that normocapnia could not be achieved by HFOV or conventional CMV without extracorporeal CO_2_ removal (Brederlau et al., 2007). A similar study also found that high frequency improved lung recruitment (Muellenbach et al., 2008). HFOV with extracorporeal membrane oxygenation (ECMO) has been shown to attenuate lung inflammation in a saline lavage pig model compared to a pressure control strategy with a V_T_ of 6 ml/kg (Muellenbach et al., 2010).

Spontaneous breathing during HFOV was evaluated using a custom demand flow valve in a saline lavage pig model of lung injury and found improved gas exchange with spontaneous breathing (van Heerde et al., 2009), possibly by shifting ventilation to more dependent lung zones (van Heerde et al., 2010). Transpulmonary pressure monitoring may help identify the lowest mPaw required to improve oxygenation and may result in fewer hemodynamic adverse effects of HFOV that occur when higher than necessary mPaw is employed (Karmrodt et al., 2006; Klapsing et al., 2018).

### Adult Evidence

#### Case Series and Observational Studies

Early case series of HFOV in adults reported improvements in oxygenation with variable effects on hemodynamics (Fort et al., 1997; Claridge et al., 1999; Mehta et al., 2001). Subsequent studies confirmed these results, but most enrolled less than 50 subjects (Fort et al., 1997; Claridge et al., 1999; Mehta et al., 2001; Andersen et al., 2002; David et al., 2003; Mehta et al., 2004; Ferguson et al., 2005; Finkielman et al., 2006; Pachl et al., 2006; Fessler et al., 2008; Kao et al., 2011; Niwa et al., 2011; Camporota et al., 2013; Jog et al., 2013; Naorungroj et al., 2015; Thind et al., 2021). HFOV has also been described in three small case series of burn patients involving 6 to 30 subjects with mortality between 32 and 83% (Cartotto et al., 2001; Cartotto et al., 2004; Cartotto et al., 2009). Two of these studies showed an improvement in arterial partial pressure of oxygen (PaO2)/FiO2 (P/F) during HFOV (Cartotto et al., 2001; Cartotto et al., 2004), but oxygenation index (OI) only improved in subjects without inhalation injury (Cartotto et al., 2009). HFOV has also been used successfully in patients with elevated intracranial pressure (David et al., 2005), chronic obstructive pulmonary disease exacerbation failing CMV (Frerichs et al., 2012), and in conjunction with a extracorporeal CO_2_ removal (Lubnow et al., 2010).

The two largest case series included 156 (Mehta et al., 2004) and 102 (Camporota et al., 2013) subjects, with mortality rates of 63 and 48%, respectively. The first, published in 2004, found improvements in oxygenation during HFOV, and that mortality was associated with delayed HFOV initiation (Mehta et al., 2004). The second was published in 2013 and showed higher survival to be associated with younger age, greater initial improvement in P/F, and lower illness severity (Camporota et al., 2013).

While most studies have set the HFOV mPaw 3–5 cmH_2_O above the CMV mPaw, others have evaluated strategies to optimize lung volumes and set optimal mPaw (Ferguson et al., 2005). The TOOLs study evaluated the combination of HFOV and recruitment maneuvers in 25 adults with early ARDS. A recruitment maneuver (40 cmH_2_O for 40 s) was performed and mPaw was increased until F_I_O_2_ was <0.60, then targeted between 30 and 22 cmH_2_O before decreasing F_I_O_2_ (Ferguson et al., 2005). This resulted in significant improvements in P/F and OI, and ICU mortality was 44%. The recruitment maneuvers were well-tolerated, with 3.3% were stopped due to hemodynamic instability (Ferguson et al., 2005). Casserly et al. evaluated a method to determine the optimal mPaw in seven subjects and assessed changes in end-expiratory lung volume by measuring chest wall dimensions (Casserly et al., 2013). After a recruitment maneuver (40 cmH_2_O for 40 s), the mPaw was set at 35 cmH_2_O for 15 min, then reduced by 2.5 cmH_2_O every 15 min until the PaO_2_ was <60 mmHg or mPaw was 15 cmH_2_O. Lung volume was found to increase in a sigmoid shape, as did PaO_2_, although PaCO_2_ had a U-shaped curve as mPaw increased (Casserly et al., 2013).

In a study of 131 subjects with 60% mortality, HFOV was associated with significant increases in fentanyl, midazolam, and cisatracurium use, but no increase in propofol use over the first 4 days (Burry et al., 2013).

An early crossover study of HFOV in 16 adults with severe ARDS found that HFOV resulted in worsening right ventricular function and decreased cardiac index once mPaw was >5 cmH_2_O above the mPaw on CMV (Guervilly et al., 2012). Another study in 12 adults with ARDS found improvement in P/F with HFOV and tracheal insufflation with no difference in cardiac index and higher central venous saturation (Vrettou et al., 2014). The relationship between mPaw and esophageal pressure has been shown to be linear and highly correlated with set mPaw (Guervilly et al., 2016).

A crossover trial evaluated short-term prone positioning during HFOV compared to supine/prone CMV. Patients undergoing CMV had the PEEP set 2 cmH_2_O above the lower inflection point of the pressure-volume curve, while those undergoing HFOV had the mPaw was set 5 cmH_2_O above the CMV mPaw. Prone positioning improved P/F in both groups, but P/F did not improve during HFOV when patients were supine. Inflammatory markers were lower in the prone HFOV group (Papazian et al., 2005). A different crossover trial evaluating HFOV plus prone positioning in 43 subjects with ARDS found that HFOV maintained lung recruitment from prone positioning, and that P/F was higher in the HFOV prone and CMV prone groups (Papazian et al., 2005).

Due to the significant improvement in oxygenation observed in case series and observational studies, there was great enthusiasm for the use of HFOV as a strategy to improve mortality. Two early RCTs evaluated the efficacy of HFOV in adult ARDS. The MOAT trial conducted between 1997 and 2000 (Derdak et al., 2002) enrolled 148 subjects with ARDS, with 75 in the HFOV group and 73 in CMV group. Groups were similar at baseline, although neither group was receiving lung protective ventilation (PIP 39 vs. 38 cmH_2_O, V_T_ 10.5 vs. 10.1 ml/kg) at enrollment. Mortality was 37% in the HFOV group and 52% in the CMV group but did not reach statistical significance. Survivors had lower OI after 24 h, regardless of group assignment (Derdak et al., 2002). Another RCT enrolled 61 subjects with ARDS from 1997–2001 (Bollen et al., 2005); it was stopped due to slow enrollment and found no difference in mortality between groups. Of note, the control group in this trial did not receive lung-protective ventilation (Bollen et al., 2005).

In 2013, the OSCAR and OSCILLATE trials were published (Ferguson et al., 2013; Young et al., 2013). The OSCAR trial randomized 397 subjects with ARDS (P/F < 200 on a minimum of 5 cmH_2_O PEEP, ventilated for <48 h) to HFOV and 398 to CMV in the United Kingdom. HFOV was set with a frequency of 10 Hz, mPaw 5 cmH_2_O above plateau pressure on CMV, bias flow 20 L/min, and an inspiratory time of 50%. Ventilation was managed to keep pH > 7.25 by maximizing the amplitude prior to adjusting the Hz, with a minimum of 5 Hz. The oxygenation target was a PaO_2_ between 60 and 75 mmHg. The CMV group was not controlled but centers were encouraged to target a V_T_ 6–8 ml/kg and use a PEEP:F_I_O_2_ table. The groups were well-matched prior to randomization. This study showed no differences in mortality or ventilator-free days between the HFOV and CMV groups (Young et al., 2013).

The OSCILLATE trial randomized 548 subjects with ARDS and a P/F ≤ 200 with an FiO_2_ ≥ 0.50 to HFOV or CMV (Ferguson et al., 2013). HFOV was managed using a recruitment maneuver (40 cmH_2_O for 40 s) at initiation, then mPaw was set at 30 cmH_2_O and subsequently adjusted using a mPaw:F_I_O_2_ table, with the F_I_O_2_ needing to be ≤0.40 before the mPaw was decreased <30 cmH_2_O. The highest frequency possible was used to maintain pH > 7.25. Groups were similar at baseline, although vasopressor use was high in both groups at enrollment. Vasopressor and neuromuscular blockage use increased over time in the HFOV group. HFOV subjects received higher mPaw throughout and had a more positive fluid balance, while CMV subjects received a V_T_ of 6.1 ml/kg and a PEEP of 18 cmH_2_O (Ferguson et al., 2013). This trial was stopped early due to higher mortality in subjects randomized to HFOV. A post-hoc analysis of 4 RCTs of HFOV found it likely to be harmful in mild to moderate ARDS but possibly beneficial in those with severe disease (P/F < 64) (Meade et al., 2017). The adult RCTs are summarized in Table 2.

**TABLE 2:** Adult randomized controlled trials.

Trial	HFOV	HFOV mPaw Initial	Hz	Amplitude	Mortality (%)	Subjects	CMV PEEP	CMV V_T_	Max plateau	Mortality (%)	Comment
MOAT Derdak et al. (2002)	75	CMV mPaw +5	5	For chest wiggle	37	73	≥10 cmH2O	10 ml/kg	None	52	No difference mortality, no lung protective ventilation in control
Bollen et al. (2005)	37	CMV mPaw +5	5	For chest wiggle	32	24	Up to 15 cmH_2_O	8–9 ml/kg	None	38	HFOV mPaw increased for lung volume and PaO2
OSCAR Young et al. (2013)	397	CMV plateau +5	10, mean 7.8 on day 1	Max, then Hz adjusted	42	398	Table	6–8 ml/kg	N.R.	41	Each site had a single HFOV vent. CMV not controlled
OSCILLATE Ferguson et al. (2013)	275	Recruitment maneuver 30 cmH2O, mPaw:FIO2 table	Highest possible	90 mbar	47	273	—	6 ml/kg	≤35 cmH2O	35	High vasopressor use, high mPaw in HFOV

### Post OSCAR and OSCILLATE

Few studies have been published after the publication of OSCAR and OSCILLATE, and there has been a significant decrease in HFOV use in clinical practice (Tatham et al., 2021). A large cohort study of rescue strategies in adult patients with severe acute hypoxemic respiratory failure found that HFOV was used in 6% of all subjects and was the second most common rescue strategy after inhaled pulmonary vasodilators (Moreno Franco et al., 2016). A survey of critical care specialists found that only 8% would consider HFOV as rescue while 26% would likely never utilize it (Alhurani et al., 2016). Additional studies have shown HFOV use to be rare in the current era, with one reporting 4.3% of rescue cases (Duan et al., 2017), another reporting 1.7% of ventilator epochs (Jabaley et al., 2018), and only 10% of ICUs in the UK reporting any HFOV use (Jha et al., 2021). In 2021, the use of HFOV was reported in a single center study of 48 subjects with a OI 36 and a mortality rate of 92% (Thind et al., 2021).

### Systematic Reviews

Multiple meta-analyses following the publication of the OSCAR and OSCILLATE trials (Ferguson et al., 2013; Young et al., 2013) concluded that HFOV does not improve mortality compared to CMV; it also showed similar risk as CMV for barotrauma or hypotension but lower rate of treatment failure (Gu et al., 2014; Huang et al., 2014; Maitra et al., 2015; Sud et al., 2016; Goligher et al., 2017). Following those trials, multiple clinical practice guidelines (CPGs) recommended against the routine use of HFOV in the adult population (Claesson et al., 2015; Cho et al., 2016; Fan et al., 2017). A CPG from the American Thoracic Society, European Society of Intensive Care Medicine, and the Society of Critical Care Medicine suggested that future research on HFOV should employ lower mPaw strategies, have HFOV settings titrated to lung mechanics, and focus on its role as a rescue therapy (Fan et al., 2017).

Non-systematic reviews have suggested HFOV has not been shown to improve outcomes due to inadequately low frequency, excessive mPaw, and the need for neuromuscular blockade (Nguyen et al., 2016). Others have suggested the failure of HFOV was related to overdistension of non-injured alveoli, transmission of high mechanical power, excessive mPaw in subjects with poor recruitability, and adverse hemodynamics, particularly right ventricular dysfunction/failure (Dreyfuss et al., 2015; Sklar et al., 2017). The resonant frequency of the lung may also factor into patient outcomes, although identifying the optimal frequency in clinical practice is still a major challenge as it will vary among patients or even within the same patient at different stages of lung disease (Sklar et al., 2017). Despite limited data, HFOV may benefit patients with P/F < 65 and should be used cautiously in subjects with significant (pH < 7.23) respiratory acidosis (Sklar et al., 2017; Fielding-Singh et al., 2018).

Despite the strong physiologic rationale for its use and reproducible improvements in gas exchange, available data has not demonstrated a major outcome benefit for the routine use of HFOV in adults. Enthusiasm for HFOV has significantly decreased following the publication of the OSCAR and OSCILLATE trials, and its use has been largely abandoned in adult patients. This is despite subgroup analyses suggesting HFOV may have of benefit in profoundly hypoxemic patients (i.e., P/F is <64) (Sklar et al., 2017; Fielding-Singh et al., 2018). These RCTs enrolled subjects with a P/F < 200, used aggressive recruitment maneuvers, and the OSCILLATE trial enrolled a large number of subjects with hemodynamic instability requiring vasopressor, along with an aggressive mPaw protocol (Ferguson et al., 2013; Young et al., 2013). It is possible that these trials enrolled subjects who were not ill enough to benefit from HFOV, or that subjects with poorly recruitable lung were harmed by the high mPaw strategy employed (both from a barotrauma and preload-dependency standpoint). No clinical trials performed to date have evaluated lung recruitability prior to enrollment. In addition, the OSCAR trial did not control CMV in the control group, only had a single ventilator available for each site, used an HFOV ventilator with the inspiratory time fixed at 50%, and staff received limited education to use the ventilator (Young et al., 2013). Importantly, the management of HFOV is complex and can be affected by the ventilator used, specific HFOV strategy, staff education, and operator familiarity with the device or strategy. Together, available data illustrate the inherent challenges of studying complex interventions such as HFOV, and the fact that optimization of HFOV settings may not have been achieved in the adult RCTs. In addition, existing studies are limited by the inherent heterogeneity of diseases and concurrent treatment strategies employed in HFOV studies.

For adult patients, it is uncertain whether additional large clinical trials will be performed. If that were the case, future trials should focus on patients with a greater disease severity (P/F < 75 or pH < 7.20) and enroll patients in units where ECMO is not readily available. Trials should also attempt to include transpulmonary pressure monitoring and electric impedance tomography, along with evaluation of lung recruitability prior to enrollment. Caution should be exercised if incorporating recruitment maneuvers into a trial protocol, as these have not been shown to improve outcomes (Pensier et al., 2019; Ibarra-Estrada et al., 2021).

### Pediatric Evidence

#### Observational Studies

Early case series in children, published prior to 2000, found improved oxygenation, with variable effects on hemodynamics and mortality rates between 0 and 48% (Arnold et al., 1993; Goodman and Pollack, 1998; Duval et al., 1999; Fedora et al., 2000; Winters et al., 2000). These studies were small, single center studies that included patients receiving HFOV for a variety of indications and disease severity. These were followed by a large, multicenter study of 290 subjects that found a mortality rate of 32% in subjects with a P/F 75–90 and an OI 27–33 (Arnold et al., 2000). Oxygenation improved during HFOV and mortality was associated immunocompromised state, OI after 24 h of HFOV, sepsis, and chronic lung disease (Arnold et al., 2000). A subsequent study of 112 subjects in the era of lung-protective ventilation was unable to identify any risk factors for mortality but also found improved oxygenation during HFOV (Babbitt et al., 2012). A study of 34 subjects identified improvement in oxygenation and organ dysfunction score as independent predictors of mortality (Chattopadhyay et al., 2020).

Children often develop acute hypoxemic respiratory failure after hematopoietic stem cell transplant and 91% of centers performing stem cell transplants used HFOV as rescue (McArthur et al., 2011). A multi-center retrospective study on the use of HFOV in 85 children following hematopoietic stem cell transplant found that those treated with HFOV were 3 times more likely to die than those who did not receive HFOV (Rowan et al., 2016). They suggested that, when considering the use of HFOV in this population, it should be initiated within 5 days of respiratory failure. Another study of children with severe PARDS following hematopoietic stem cell transplantation found that HFOV use was associated with an odds ratio of 6.28 (95% confidence interval 1.16–34.12) for death (van Gestel et al., 2008).

A secondary analysis of the PARDIE study dataset evaluating rescue strategies for severe hypoxemia found that nearly all centers had HFOV available and used it in 9.3% of subjects (Khemani et al., 2019; Rowan et al., 2020). This rate was similar to the use of prone positioning (10%), slightly lower than inhaled nitric oxide (13%) but higher than ECMO (3%) (Rowan et al., 2020). HFOV was used more frequently in middle income countries, in patients with higher illness severity, and immunocompromised patients.

The feasibility of a physiologic, open-lung recruitment strategy of HFOV was described in 115 subjects treated in a single center in the Netherlands (de Jager et al., 2019). The HFOV strategy consisted of a starting frequency of 12 Hz for all subjects and used incremental-decremental staircase adjustments to select the “optimal” mPaw on the deflation limb of pressure-volume curve (de Jager et al., 2019). Ventilation was controlled by adjusting the frequency, but only after maximizing the power (amplitude) setting (de Jager et al., 2019). The reported mortality for different PARDS severity was similar to the mortality reported in the PARDIE study (Khemani et al., 2019). A different report from the same group found minimal hemodynamic effects, with 88% sensitivity and 54% specificity for changes in lung volumes (de Jager et al., 2020). This is the HFOV strategy currently being investigated as part of the PROSPECT trial (https://prospect-network.org/).

The first pediatric RCT of HFOV, published in 1994, enrolled 70 children ≤35 kg with an OI > 13, acute diffuse lung injury, or barotrauma (Arnold et al., 1994); it showed significant improvements in oxygenation compared to CMV, but no difference in survival (59% for CMV vs. 66% for HFOV). The OI was the strongest predictor of mortality with an OR of 20.8 (95% confidence interval 3.4 to 128.4, *p* < 0.001)and HFOV subjects were less likely to require oxygen at discharge (Arnold et al., 1994).

More recently, three propensity score matched analyses of HFOV compared to CMV have been published. The first used the Virtual PICU Systems database and compared early vs. late HFOV to CMV (Gupta et al., 2014). Subjects were matched for age, weight, CPR, severity of illness, ECMO, dialysis, arterial catheter, central access, hemodynamics, diagnoses. This study found that HFOV was associated with an increased length of MV, ICU length of stay, and mortality (Gupta et al., 2014). Importantly, this database did not collect crucial variables such as ventilator settings, gas exchange, and measures of oxygenation. As such, the adequacy of propensity matching (and, thus, the study findings) should be taken with caution. A reanalysis of the RESTORE trial dataset used propensity score matching to compare early HFOV with CMV or late HFOV (Bateman et al., 2016) and found no significant association with mortality; however, secondary analyses revealed early HFOV was associated with a higher mortality after accounting for risk category, with the 2 highest risk groups having increased mortality. Subjects in the HFOV group spend more time on the ventilator (Bateman et al., 2016). HFOV use increased as OI increased, with subjects more likely to be placed on HFOV once OI was >8, and those with an OI ≥ 40 were 17 times more likely to be placed on HFOV (Bateman et al., 2016). The Pediatric Acute & Critical Care Medicine Asian Network (PACCMAN) group performed a propensity matched study using a large multicenter database of pediatric ARDS and found that, compared to CMV, HFOV was associated with increased mortality and fewer ICU free days, but no difference for ventilator free days (Wong et al., 2020).

These studies are significantly limited as most did not record granular respiratory details such as plateau pressure, V_T_, driving pressure, and rationale for starting HFOV. Despite efforts to control for illness severity, it is likely that patients receiving HFOV were in fact sicker or failing CMV prior to placement on HFOV, thus resulting in mismatched acuity between the groups. Importantly, the presence of shock, vasopressor use, and renal failure were not included in the models.

Three additional RCTs have been published in recent years. A small trial, published in 2016, randomized 18 children with severe ARDS to HFOV (*n* = 9) or CMV (*n* = 9) with lung recruitment maneuvers. HFOV improved oxygenation and was well-tolerated hemodynamically. The overall survival was 89%, with one death in each group. Of note, 3 (33%) subjects randomized to the CMV group crossed over to HFOV (Samransamruajkit et al., 2016). The second trial, published in 2017, compared HFOV to protective CMV in 200 subjects with pediatric ARDS (El-Nawawy et al., 2017). HFOV resulted in improved oxygenation and more rapid increase in P/F but no differences in mortality (43% for CMV vs. 45% in HFOV), days of MV, OI difference after 24 h, and PICU length of stay (El-Nawawy et al., 2017).

A RCT of 61 infants with ARDS after high-risk atrial septal defect or ventricular septal defect repair compared HFOV to CMV; both groups also received surfactant replacement therapy (Zheng et al., 2021). The primary outcome was improvement in arterial blood gases. CMV strategy called for an inverse I:E ratio, with PIP 18–25 cmH_2_O and PEEP 4–-6 cmH_2_O but actual values were not reported. HFOV resulted in relatively small differences in PaO_2_, P/F, PaCO_2_, and OI. The HFOV group had shorter time on mechanical ventilation, ICU length of stay, and total hospital length of stay (Zheng et al., 2021). Pediatric RCTs are summarized in Table 3.

**TABLE 3:** Pediatric randomized controlled trials.

Trial	HFOV	HFOV mPaw Initial	Hz	Amplitude	Mortality	Subjects	CMV PEEP	CMV V_T_	Max plateau	Mortality	Comment
Arnold et al. (1994)	29	CMV mPaw + 4–8	5–10	For chest wiggle	66%	29	Increased for oxygenation	10 ml/kg	None	59%	Control group did not receive LPV, PIP >40 at baseline in both groups
Samransamruajkit et al. (2016)	9	CMV mPaw + 5–8	5	3x CMV mPaw	NR	9	RM followed by decremental PEEP maneuver	6–8 ml/kg	None	NR	89% overall survival, between groups not reported
El-Nawawy et al. (2017)	100	CMV plateau + 3–5	5–12	1.-5-3 ml/kg	45%	100	NR	5–8 ml/kg	PIP ≤35	43%	No difference in PICU LOS, OI at 24 h 50% I:E
Zheng et al. (2021)	31	10–15, with slow recruitment maneuver	8–12	30–40	3.2%	30	4–6	NR	PIP ≤30	10%	Congenital heart disease, also received surfactant replacement, shorter time on MV in HFOV group
											CMV group received inverse I:E ventilation

A recent systematic review of pediatric RCTs, propensity score matched studies, and observational studies failed to show an advantage of HFOV over CMV, with no demonstrable reduction in mortality, time on MV, or barotrauma (Junqueira et al., 2021). Of note, the GRADE certainty was low or very low for all studied outcomes (Junqueira et al., 2021). Recent non-systemic reviews suggest that, while HFOV strategies still require refinement, HFOV remains a viable rescue therapy for severe pediatric ARDS (Moerer et al., 2017; Nardi et al., 2017; Ng and Ferguson, 2017). Kneyber et al. advanced that HFOV was not optimized in prior RCTs (Kneyber et al., 2012) and suggested starting HFOV if SpO_2_ <88%, PaO_2_ < 50 mmHg with an F_I_O_2_ > 0.60 on sufficient support or in the presence of refractory respiratory acidosis. The suggested strategy involves increasing mPaw using incremental mPaw steps while following an expected rise in SpO2 until overdistension is observed. This is followed by a stepwise reduction in mPaw until the point of derecruitment, then the mPaw is set 2–4 cmH_2_O above this decruitment point (Kneyber et al., 2012). This strategy also advocates the use of the highest tolerable frequency (Hz) to minimize V_T_, and is currently being investigated in the PROSPECT trial (https://prospect-network.org/).

Similar to adults, available data do not support the use of HFOV in children with severe ARDS, despite consistently observed improvement in oxygenation. Unlike adults, however, these data are significantly limited by low quality RCTs and HFOV is still widely utilized in pediatric ICUs. Hopefully, the ongoing PROSPECT trial will provide more definitive answers on the role of HFOV in pediatric ARDS. Beyond the PROSPECT trial, the use of HFOV as a rescue strategy should be investigated, perhaps through a large registry or database that includes granular variables to allow for improved patient-level matching of relevant characteristics. Physiologic studies should include electric impedance tomography to evaluate the continuous relationship between lung volume and gas exchange. We await the results of the PROSPECT trial, an international multicenter, two-by-two factorial, response-adaptive RCT evaluating CMV and HFOV, along with prone and supine positions in children with severe hypoxemic respiratory failure.

### Effect of HFOV on the Right Ventricle and Hemodynamics

Positive pressure ventilation can have negative effects on right ventricular (RV) function and overall hemodynamics, with some investigators suggesting this as a possible reason why RCTs of HFOV have failed to show an outcome benefit (Dreyfuss et al., 2015; Sklar et al., 2017). When lung volume is excessively increased, there is a potential for an increase in West zone 1 (ventilation with no perfusion) lung units, which results in higher RV afterload from an increase in pulmonary vascular resistance (PVR). Conversely, the underinflated lung (i.e., below functional residual capacity), also lead to increased PVR and increased RV afterload (Simmons et al., 1961). Thus, the use of high mPaw during HFOV may be detrimental to RV function due to increased PVR. Available data, however, do not show significant hemodynamic effects during stepwise recruitment and de-recruitment maneuvers (de Jager et al., 2020). Additionally, the elevated mPaw used during HFOV may adversely affect preload and result in the need for intravascular fluid expansion that can lead to volume overload.

An observational study found that, when HFOV was initiated with a mPaw 5 cmH_2_O above CMV mPaw, no differences in mean arterial pressure or heart rate were noted, but right atrial pressure increased, cardiac index slightly decreased, and left ventricular end-diastolic pressure decreased in a study of nine subjects with ARDS (David et al., 2004). Another study found HFOV did not appear to have a large effect on left and right ventricular function, but the cardiac index decreased by 13% when HFOV mPaw was set 5 cmH_2_O above the CMV mPaw (Ursulet et al., 2015). Additional studies found no association between body mass index and mortality, and that the higher mortality observed in the OSCILLATE was not related to hemodynamic changes 2 h after HFOV initiation (Tlayjeh et al., 2019; Angriman et al., 2020). One study found that an acute cor pulmonale score ≥2 and a P/F ≥ 100 were directly associated with mortality during HFOV (Angriman et al., 2020).

### Staff Education and Competency

Management of HFOV is complex and requires advanced skills, device specific training/competency, physiologic understanding, and critical thinking as the learning curve for HFOV is steep. HFOV management is challenging even for teams experienced in its use. Data evaluating HFOV education are sparse. Deficits in basic MV management and assessment for asynchrony by critical care physicians has been reported (Colombo et al., 2011). A narrative review concluded that there is a paucity of information describing MV education in graduate medical education (Keller et al., 2019). Likewise, there is a dearth of guidance to facilitate staff education and verify competency of the end user. Simulation based training has shown promise in improving the outcome of learners as an addition to traditional didactic teaching (Cook et al., 2011). High-fidelity simulation has been shown to improve knowledge and skills related to MV in anesthesiology residents (Spadaro et al., 2017). There remains no standard approach to teach HFOV management nor to assess staff competency.

Staff education may be an underappreciated factor in prior clinical trials, particularly the OSCAR trial in which a new HFOV ventilator was used and some centers may have had limited experience with HFOV (Young et al., 2013). Education is even more critical when complex maneuvers, such as dynamic sustained inflation for lung recruitment or staircase titration of mPaw, are used to determine optimal mPaw (de Jager et al., 2019). Training and education in the use of HFOV settings, such as amplitude, require the user to assess subjective parameters such as “chest wiggle” as a surrogate for appropriate ventilation (Meyers et al., 2019). Inexperienced team members may have difficulty properly assessing the degree of chest wiggle or how to react appropriately, as frequency adjustments are counter-intuitive compared to CMV. Better feedback and assessment tools are needed to guide learning objectives and determine end-user competency. Future exploration into teaching and training methods utilizing HFOV are warranted; in the meantime, yearly education and competency assessment for centers that do not routinely utilize HFOV is suggested (Thind et al., 2021).

## Conclusion

HFOV has largely been abandoned in adults following two large clinical trials despite a strong physiologic rationale and promising animal data. Available data do not support its routine use in adult or pediatric ARDS but it may have utility in more severe disease and pediatric data are of low quality. The mode is complex and patient outcomes may be affected by the ventilator used, HFOV strategy, and staff education.
